# Micro-CT Assessment of Dentin Mineral Density and Furcation Morphology in Primary First and Second Molars

**DOI:** 10.3390/dj14080473

**Published:** 2026-08-03

**Authors:** Maria Kirilova, Ralitsa Bogovska-Gigova, Zdravka Yaneva, Krasimir Hristov

**Affiliations:** Department of Pediatric Dentistry, Faculty of Dental Medicine, Medical University Sofia, 1000 Sofia, Bulgaria; m.kirilova@fdm.mu-sofia.bg (M.K.); z.yaneva@fdm.mu-sofia.bg (Z.Y.); k.christov@fdm.mu-sofia.bg (K.H.)

**Keywords:** primary molars, furcation area, dentin mineral density, micro-CT, furcation morphology, accessory canals

## Abstract

**Background/Objectives:** The furcation region of primary molars is a critical anatomical zone with high clinical relevance in endodontics and restorative dentistry due to its proximity to the pulp chamber and potential accessory canal communications. This study aimed to quantitatively evaluate and compare dentin mineral density in the crown and furcation regions, as well as furcation structure thickness, maximum dentin thickness, and volume, between primary first and second molars using micro-computed tomography. **Methods:** Thirty-eight extracted primary molars (19 first and 19 second molars) with physiological root resorption affecting less than two-thirds of the root length were included. Teeth were scanned using a SkyScan 1272 micro-CT system. Mineral density was calibrated using 0.3 and 1.25 g/cm^3^ hydroxyapatite phantoms. Morphometric parameters of the furcation dentin were analyzed with CTAn software. Statistical analysis was performed using non-parametric tests (*p* < 0.05). **Results:** Dentin mineral density was significantly lower in the furcation region compared to the crown in both first (median: 1.64 g/cm^3^, IQR: 1.62–1.68 vs. 1.84 g/cm^3^, IQR: 1.82–1.90) and second molars (1.71 g/cm^3^, IQR: 1.70–1.73 vs. 1.96 g/cm^3^, IQR: 1.93–1.96) (*p* < 0.001). Second primary molars showed significantly higher mineral density, greater furcation structure thickness (1.50 mm vs. 1.30 mm), maximum dentin thickness (1.86 mm vs. 1.53 mm), and furcation dentin volume (80.8 mm^3^ vs. 46.2 mm^3^) than first molars (all *p* < 0.05). Accessory canals were detected in 15.79% of specimens. **Conclusions:** This study demonstrates significantly lower mineralization in the furcation region compared to coronal dentin in primary molars, with first molars exhibiting lower mineral density and thinner furcation structure than second molars. These anatomical and compositional differences highlight the furcation area as a vulnerable zone and suggest the potential value of considering tooth-specific approaches in clinical management. Further clinical studies are warranted to evaluate the implications for treatment outcomes.

## 1. Introduction

Primary molar anatomy demonstrates significant structural heterogeneity that has profound implications for clinical practice, particularly in endodontic and restorative dentistry. The furcation region represents a critical anatomical zone where structural integrity directly influences treatment outcomes, yet this area remains incompletely characterized in terms of mineral composition and morphometric properties [1].

Understanding regional variations in dentin mineralization is essential because mineral density correlates with mechanical properties and resistance to caries progression. Studies have demonstrated that dentin hardness and elastic modulus decrease with proximity to the pulp, with values near the pulp wall (0.52 ± 0.24 GPa hardness) significantly lower than those near the dentinoenamel junction (0.91 ± 0.15 GPa) [2]. This gradient in mechanical properties reflects underlying differences in mineral content and crystallinity, with decreased mineral content and increased carbonate content associated with reduced hardness [3].

Primary molars exhibit distinct anatomical features that differentiate them from permanent teeth and create unique clinical challenges. The proportionally larger pulp chamber, prominent pulp horns, and reduced dentin thickness between the occlusal surface and pulp chamber (approximately 4 mm from cusp tip to the roof of the pulp chamber) provide limited protection against bacterial invasion [1,4]. Importantly, the average distance between the pulpal floor and the furcation area is just 1.7 mm, which greatly increases the likelihood of furcation involvement either as a disease advances or due to accidental perforation during treatment [4].

The furcation region presents additional complexity due to the presence of accessory canals in 42.7% of primary molars, which provide pulpal-periodontal communications with important clinical implications [5,6]. These accessory canals, together with regional differences in dentin mineral density and potentially increased permeability of the pulpal floor due to lower mineralization, may facilitate the spread of infection from the pulp chamber to the interradicular area. This combination of anatomical and compositional factors may explain why primary teeth demonstrate more extensive enamel and dentin destruction from caries compared to permanent teeth, with increased peritubular dentin alterations and less effective reactive dentin formation [7].

Significant morphological and volumetric differences exist between primary first and second molars. Maxillary second molars demonstrate the highest pulp chamber volumes, while mandibular first molars show the highest risk of perforation in the furcation area [1]. Root canal morphology varies considerably, with the number of canals ranging from three to four, and maxillary molars more commonly exhibiting one-canal rather than two-canal roots [8]. The palatal root of maxillary molars is typically the longest, while the distobuccal root is the shortest [8]. These anatomical variations extend to the furcation region, where structural differences between tooth types may reflect developmental patterns and functional demands. Second molars, being larger and erupting later, may undergo more extensive post-eruptive maturation, potentially explaining the differences in mineralization patterns. Primary teeth accumulate fluoride and become harder and less porous over time through post-eruptive maturation, though they remain more caries-prone than permanent teeth throughout their lifespan [9].

The furcation region is particularly vulnerable to pathological involvement in primary molars with extensive caries. Furcation lesions represent a significant clinical challenge, with treatment success dependent on multiple factors including the extent of bone loss, presence of accessory canals, and the ability to adequately seal the pulpal floor [10]. Studies comparing different base materials for primary teeth with furcation lesions have shown that lesions heal significantly faster when mineral trioxide aggregate is used on the pulpal floor following root canal treatment [10]. The complex interradicular anatomy, including the presence of interradicular canals and diverticula in up to 10% of molars, can compromise endodontic treatment success if adequate chemical debridement and disinfection are not achieved [11]. Furthermore, only 7% of accessory canals in the furcation region are patent, suggesting that other factors, such as regional variations in mineral density, may play a greater role in the transmission of infection to the interradicular area [6].

Regional variations in dentin mineral density have direct implications for caries progression, treatment planning, and restoration success. Primary tooth enamel has a lower degree of mineralization and is more hydrophilic than permanent tooth enamel, resulting in bacterial adhesion energies approximately 10 times higher for Streptococcus mutans [12]. This increased susceptibility, combined with thinner enamel and dentin layers, accelerates the pathway to pulpal involvement [9].

The mineral content of dentin influences its response to caries and subsequent treatment. Studies using microcomputed tomography have demonstrated that different treatment protocols result in varying degrees of dentin mineralization, with hypermineralized areas observed underneath certain restorations [13]. Managing carious lesions with appropriate protocols can result in higher mineralized dentin, potentially improving long-term outcomes [13,14].

Despite the clinical importance of furcation anatomy in primary molars, comprehensive characterization of regional mineral density variations remains limited. While studies have examined overall dentin composition and mechanical properties in primary teeth, detailed analysis of mineral density differences between anatomical regions (crown versus furcation) and between tooth types (first versus second molars) has not been systematically investigated [2,15,16]. Understanding these variations is critical because reduced mineralization in the furcation region may contribute to increased susceptibility to caries progression, compromised mechanical support during restorative procedures, and higher risk during endodontic treatment. Furthermore, differences in furcation morphology and mineral density between first and second molars may necessitate tooth-specific treatment approaches and inform clinical decision-making regarding treatment selection and prognosis.

Despite the well-documented anatomical complexity of primary molars, comprehensive quantitative data on regional dentin mineral density variations—particularly comparing the crown and furcation areas—and detailed three-dimensional morphometric characterization of the furcation dentin between first and second primary molars remain limited. While previous studies have described overall pulp chamber morphology, root canal configurations, and the presence of accessory canals, calibrated mineral density measurements and combined morphometric analysis of the furcation region using high-resolution micro-CT have not been systematically performed. The present study addresses these gaps.

The aim of this study was to quantitatively evaluate and compare the mineral density of dentin in the crown and furcation regions, as well as the furcation structure thickness, maximum dentin thickness, and volume, between primary first and second molars using micro-computed tomography (micro-CT). The study tested two null hypotheses: (1) there is no significant difference in dentin mineral density between the coronal and furcation regions within primary molars; and (2) there is no significant difference in furcation dentin mineral density or morphometric parameters (structure thickness, maximum thickness, and volume) between primary first and second molars.

## 2. Materials and Methods

### 2.1. Sample Size Calculation

Sample-size calculation was performed using G*Power software (version 3.1.9.7, Heinrich Heine University, Duesseldorf, Germany). Because the study was designed as a preliminary exploratory investigation and sufficiently comparable published quantitative data were unavailable to derive a reliable empirical effect-size estimate, a large effect size was assumed for the sample-size calculation. It was determined that a total sample size of *n* = 38 was sufficient to detect a large effect size (f = 1.1) with a statistical power (1-β error) of 0.95 (95%) and a significance level (α error) of 0.05 (5%) for the Wilcoxon–Mann–Whitney test family.

### 2.2. Sample Preparation

This study included a total of 38 extracted primary molars, comprising 19 first primary molars (10 maxillary and 9 mandibular) and 19 second primary molars (11 maxillary and 8 mandibular), obtained from 38 children aged between 6 and 9 years. All teeth exhibited physiological root resorption affecting less than two-thirds of the root length (mean resorption approximately 35–40% of root length, with no involvement of the furcation area). Teeth were excluded if they presented with restorations, dental caries, advanced root resorption exceeding two-thirds of the root length, resorption involving the furcation area, or evidence of intracanal resorption.

All selected teeth were extracted solely for orthodontic purposes. Prior to extraction, informed consent was obtained from the parents/legal guardians. The study protocol was conducted in accordance with the Declaration of Helsinki and approved by the Ethics Committee of the Medical University Sofia—KENIMUS (Approval Number: 14/28.09.2023).

Following extraction, the teeth were thoroughly cleaned of soft tissue and debris. They were then stored in a 0.1% thymol solution at 4 °C. The storage time before micro-CT scanning ranged from 2 to 12 weeks. This storage protocol was chosen to prevent microbial growth while preserving the structural and mineral properties of the dental tissues.

### 2.3. Micro-CT Scanning Parameters and Image Analysis

Micro-computed tomography was used as a non-destructive, non-invasive imaging technique to evaluate dental hard tissues [17,18]. All specimens were scanned using a SkyScan 1272 desktop X-ray micro-computed tomography system (Bruker, Antwerp, Belgium). Each sample was mounted on a sample holder with the long axis of the tooth perpendicular to the horizontal scanning platform to achieve consistent positioning during image acquisition. Scanning was performed using a 1 mm aluminium filter, X-ray tube voltage of 87 kV, and a full 360° rotation with a rotation step of 0.4°. Frame averaging was performed 4 times to improve signal-to-noise ratio. The X-ray source current was set at 114 μA, corresponding to a maximum source power of 10 W. A conical beam geometry with a voxel size of 15 μm was used, enabling high-resolution imaging of the entire crown. Approximately 1130 slices were acquired per sample, with a scan time of about 20 min per tooth.

**Figure 1:**
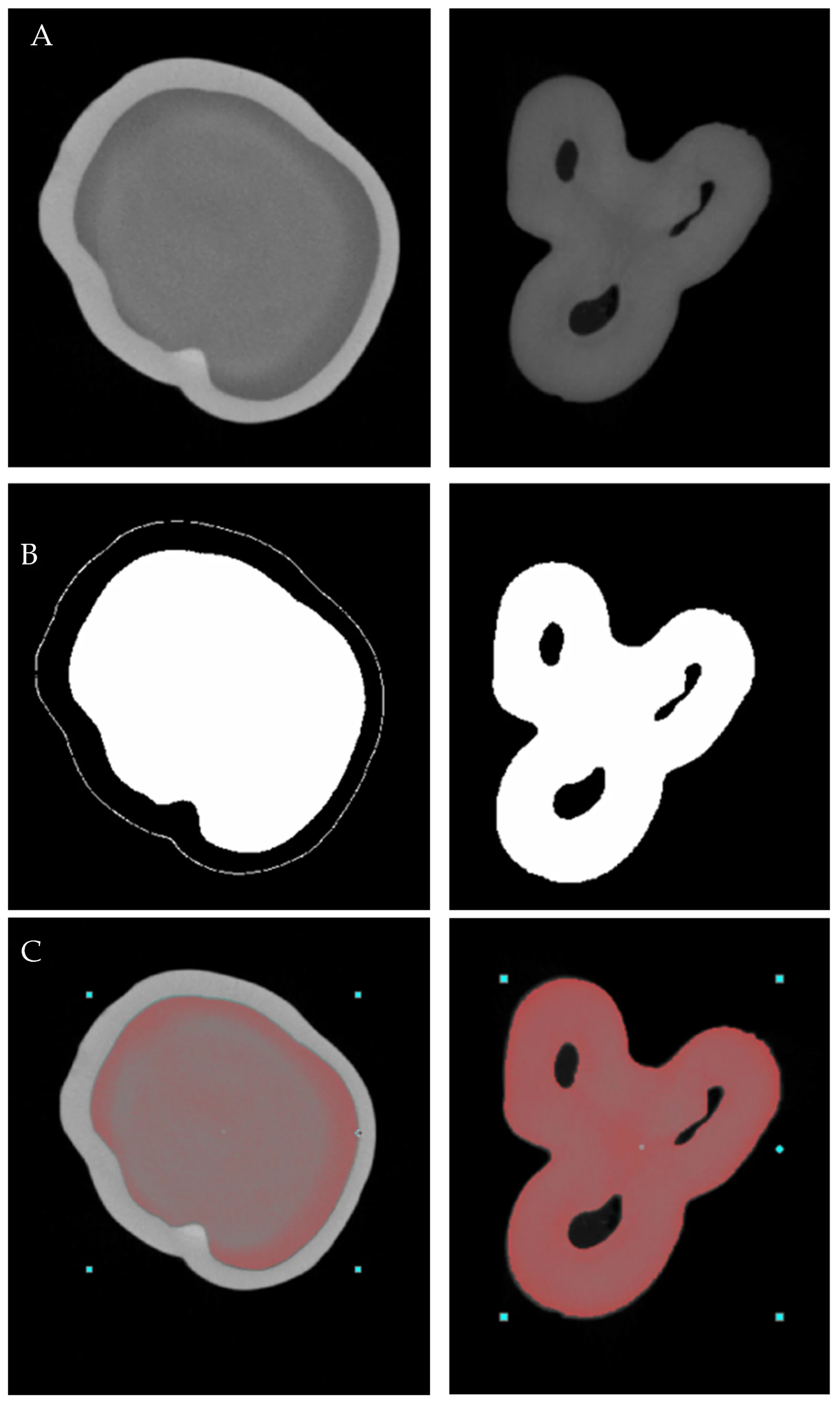

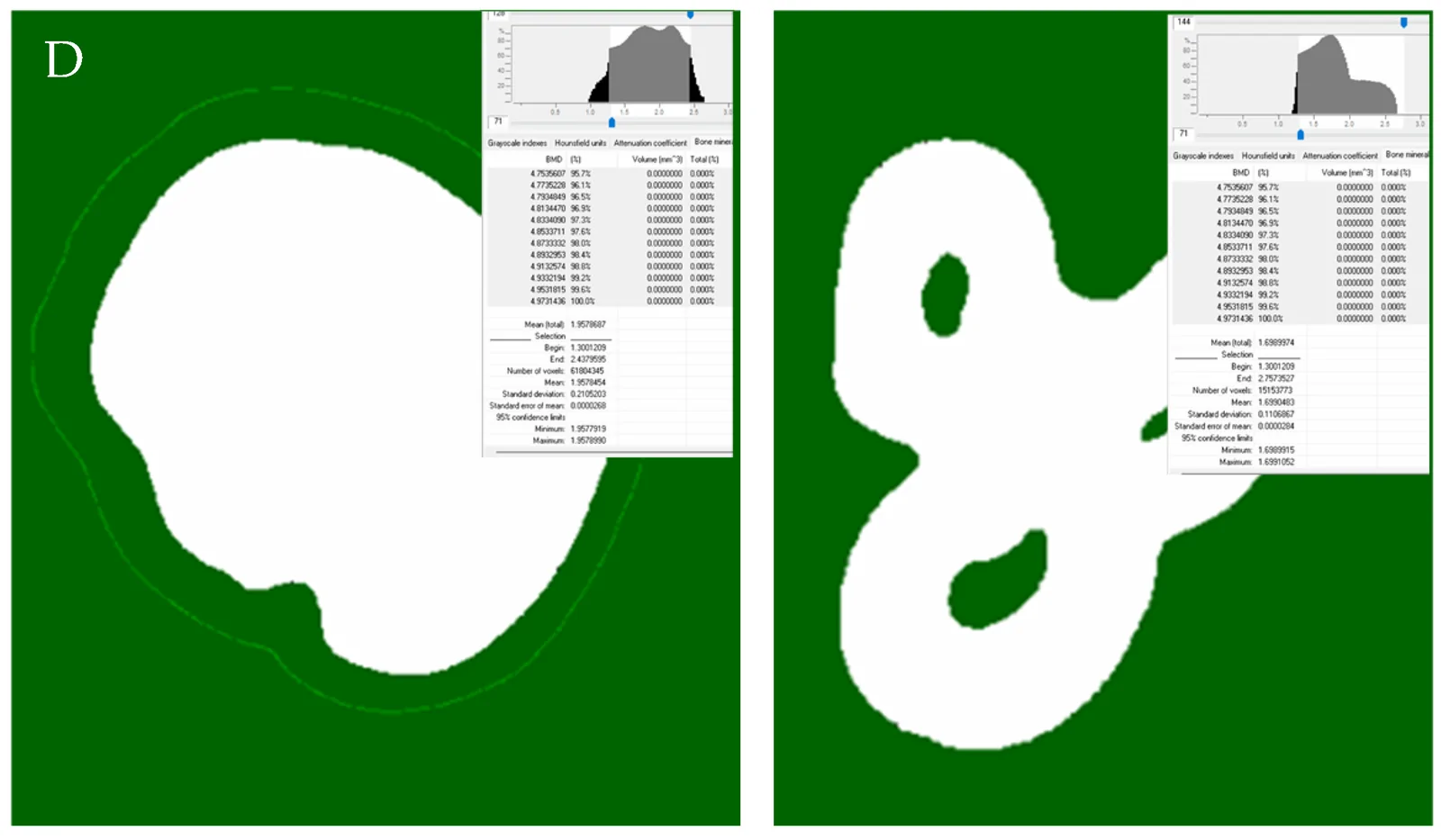
Working protocol for mineral density assessment of coronal dentin of second primary molars and furcation area of first primary molars. (**A**) Raw images, (**B**) binarization, (**C**) selecting Region of Interest (ROI), (**D**) measuring mineral density.

The tooth projections were converted to cross-sectional slices by stacking a series of two-dimensional images using SkyScan NRecon software (version 2.2.0.6, Bruker, Kontich, Belgium), with GPU acceleration. Adjustments included ring artifact reduction at level 4, beam hardening correction set to 55%, and post-scan corrections for slow changes in geometry. Hydroxyapatite calibration phantoms with nominal mineral densities of 0.3 and 1.25 g/cm^3^ were scanned using the same acquisition and reconstruction parameters as the tooth specimens. Mean reconstructed grayscale values obtained from the phantoms were used to establish a linear calibration relationship between X-ray attenuation and nominal hydroxyapatite mineral density. Accordingly, the reported values should be interpreted as micro-CT-derived hydroxyapatite-equivalent mineral-density estimates rather than direct measurements of absolute physical density. Segmentation and ROI definition were conducted in CTAn software (version 1.23.01, Bruker, Kontich, Belgium). The coronal dentin ROI was defined as the dentin volume extending from the cusp tips to the last slice immediately coronal to the pulp chamber floor, identified by its first appearance in the reconstructed slices. The furcation dentin ROI was defined as the dentin volume starting from the first slice in which the floor of the pulp chamber was clearly visible and extending apically to the first slice showing complete separation of all roots. Global thresholding was applied after visual inspection of the histogram to segment dentin from pulp and surrounding structures. The mineral density (MD, g/cm^3^, Figure 1) of the dentin, furcation structure thickness (average thickness of a segmented structure in a 3D dataset) and volume were calculated using CTAn analysis software (version 1.23.01, Bruker, Kontich, Belgium). The presence of accessory canals and the maximum thickness of the dentin in furcation area were assessed by observing and manually measuring the area with CTAn analysis software.

One examiner performed all measurements. Ten randomly selected samples were re-evaluated by the same examiner after a two-week interval. Intraclass correlation coefficients (ICCs) were calculated for mineral density, furcation volume, structure thickness, and maximum dentin thickness measurements, while Cohen’s kappa coefficient was used for accessory canal identification. ICC values ranged from 0.91 to 0.95, and kappa agreement was 0.87, indicating excellent intra-observer reliability.

### 2.4. Statistical Analysis

Statistical analysis was performed using SPSS v.19.0 (SPSS Inc., Chicago, IL, USA). Data normality was tested with the Shapiro–Wilk test; as normality was not met, non-parametric tests were used. The Wilcoxon signed-rank test was used to compare mineral density between the crown and furcation regions within each molar group because these measurements were obtained from the same specimens. The Mann–Whitney U test was used to compare independent groups of first and second primary molars with respect to mineral density, furcation structure thickness, maximum furcation dentin thickness, and furcation dentin volume. Effect sizes and 95% confidence intervals (CIs) were calculated to quantify the magnitude and precision of the observed differences. The significance level was set at *p* = 0.05.

## 3. Results

Table 1 summarizes the mineral density and morphometric findings, including median values and interquartile ranges, rank-biserial correlation coefficients, 95% confidence intervals (CIs) for the estimated differences, and corresponding *p*-values.

**Table 1:** Mineral density and morphometric parameters of the furcation region in first and second primary molars (median and interquartile range).

Parameter	First Primary Molars	Second Primary Molars	Rank-Biserial Correlation	95% CI	*p*-Value
Crown dentin mineral density (g/cm^3^)	1.84 (1.82–1.90)	1.96 (1.93–1.96)	0.90	0.1–0.15	*p* < 0.001
Furcation dentin mineral density (g/cm^3^)	1.64 (1.62–1.68)	1.71 (1.70–1.73)	0.90	0.04–0.1	*p* < 0.001
Furcation structure thickness (mm)	1.30 (1.24–1.33)	1.50 (1.46–1.55)	0.95	0.15–0.24	*p* < 0.001
Maximum dentin thickness (mm)	1.53 (1.51–1.62)	1.86 (1.76–1.96)	0.91	0.13–0.53	*p* = 0.003
Furcation dentin volume (mm^3^)	46.2 (38.4–46.7)	80.8 (72.9–84.5)	1.00	30.9–38.3	*p* < 0.001
Furcation vs crown mineral density, first molar	-	-	−1.00	0.15–0.24	*p* < 0.001
Furcation vs crown mineral density, second molar	-	-	−1.00	0.24–0.26	*p* < 0.001
Accessory canals, *n*	4 (3 lower, 1 upper)	2 (1 upper, 1 lower)	-	-	-

**Figure 2:**
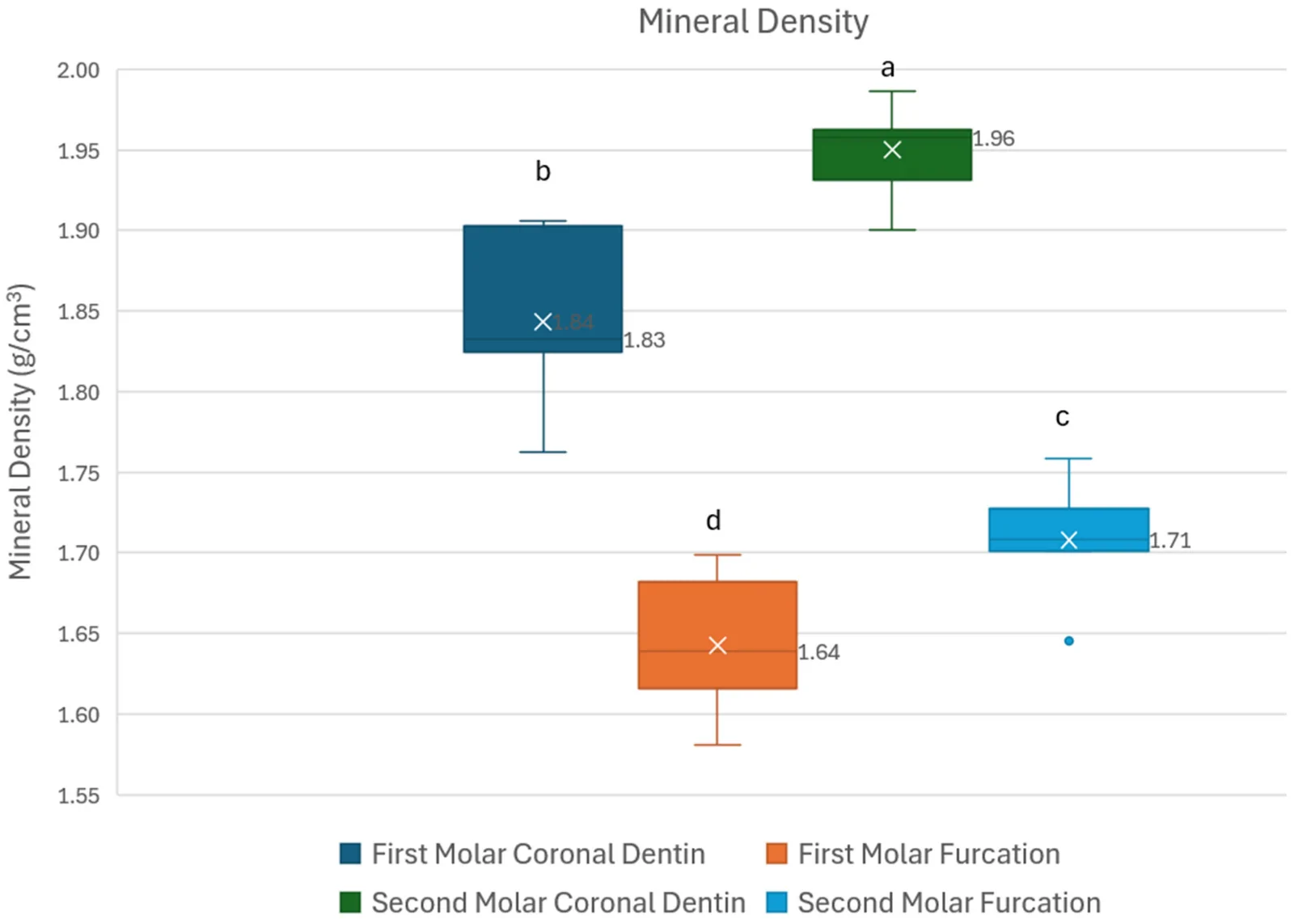
Mineral density in the crown and furcation regions of the first and second primary molars (Wilcoxon signed-rank test and Mann–Whitney U-test; different lower-case letters indicate statistical significance (*p* < 0.05)).

The results of the furcation and coronal dentin mineral density of the examined specimens are presented in Figure 2.

Wilcoxon signed-rank tests compared dentin mineral density between the furcation and crown regions within the same tooth group. For the first primary molar, coronal dentin measurements were significantly higher than furcation area (W = 0.00, *p* < 0.001), with median values of 1.84 g/cm^3^ (IQR 25th–75th percentile: 1.82–1.90 g/cm^3^) and 1.64 g/cm^3^ (IQR: 1.62–1.68 g/cm^3^), respectively. All paired observations showed lower mineral density in the furcation region than in the corresponding coronal region (r_rb_ = −1.00, 95% CI [0.15–0.24]). Similarly, for the second primary molar, coronal dentin mineral density was significantly higher than the dentin in the area of the furcation (W = 0.00, *p* < 0.001), with median values of 1.96 (IQR: 1.93–1.96 g/cm^3^) and 1.71 (IQR: 1.70–1.73 g/cm^3^), respectively. There was complete directional separation, with every second primary molar exhibiting lower mineral density in the furcation region than in its corresponding coronal region (r_rb_ = −1.00, 95% CI [0.24–0.26]). Overall, these findings demonstrate a consistent and statistically significant increase in mineral density from the furcation to the coronal level in both groups of molars. The Mann–Whitney U-test was used to compare dentin mineral density between the two groups of teeth. Second primary molars demonstrated significantly higher mineral density than first molars in both evaluated regions (*p* < 0.001), suggesting a more densely mineralized dentin structure. The differences in mineral density between first and second primary molars were consistent across both anatomical regions (r_rb_ = 0.90, 95% CI [0.1–0.15] for coronal dentin and r_rb_ = 0.90, 95% CI [0.04–0.1] for furcation area).

**Figure 3:**
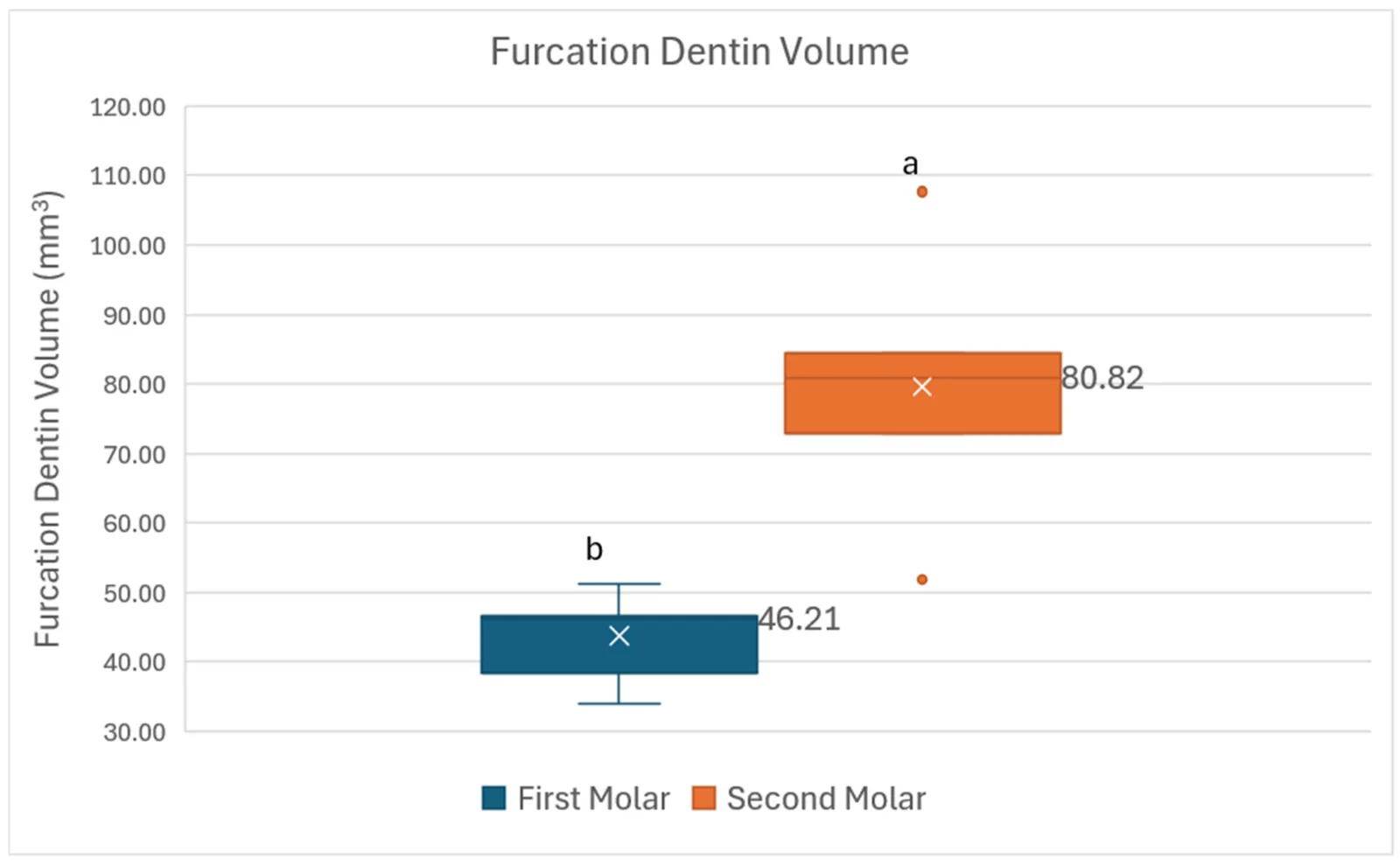
Comparison of furcation dentin volume in first and second primary molars (Mann–Whitney U-test; different lower-case letters indicate statistical significance (*p* < 0.05)).

**Figure 4:**
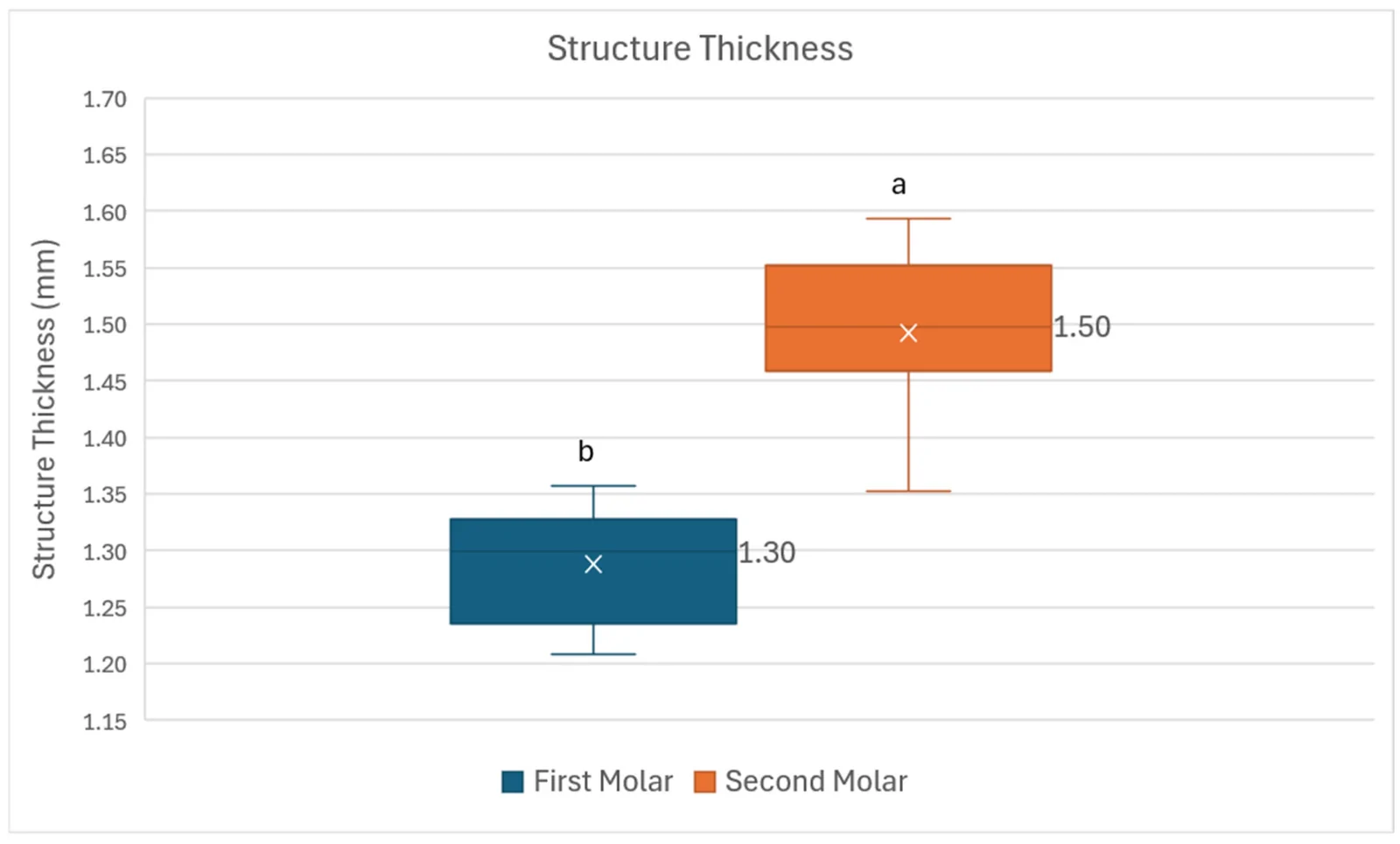
Structure thickness of first and second primary molar furcation dentin (Mann–Whitney U-test; different lower-case letters indicate statistical significance (*p* < 0.05)).

**Figure 5:**
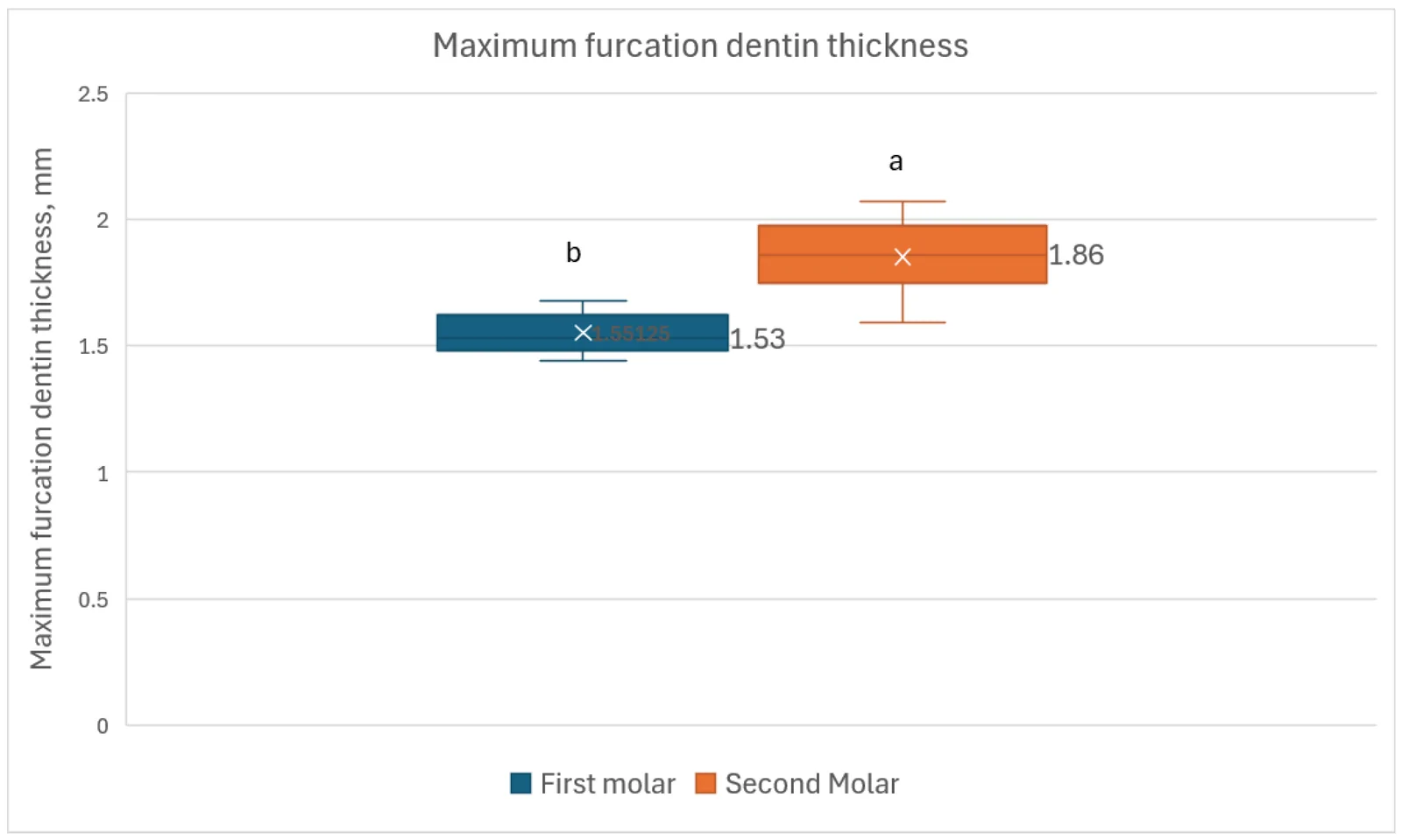
Maximum thickness of the dentin in the furcation area of first and second primary molars (Mann–Whitney U-test; different lower-case letters indicate statistical significance (*p* < 0.05)).

Figure 3, Figure 4 and Figure 5 demonstrate the dentin volume, structure thickness and the maximum dentin thickness of the primary molar furcations.

**Figure 6:**
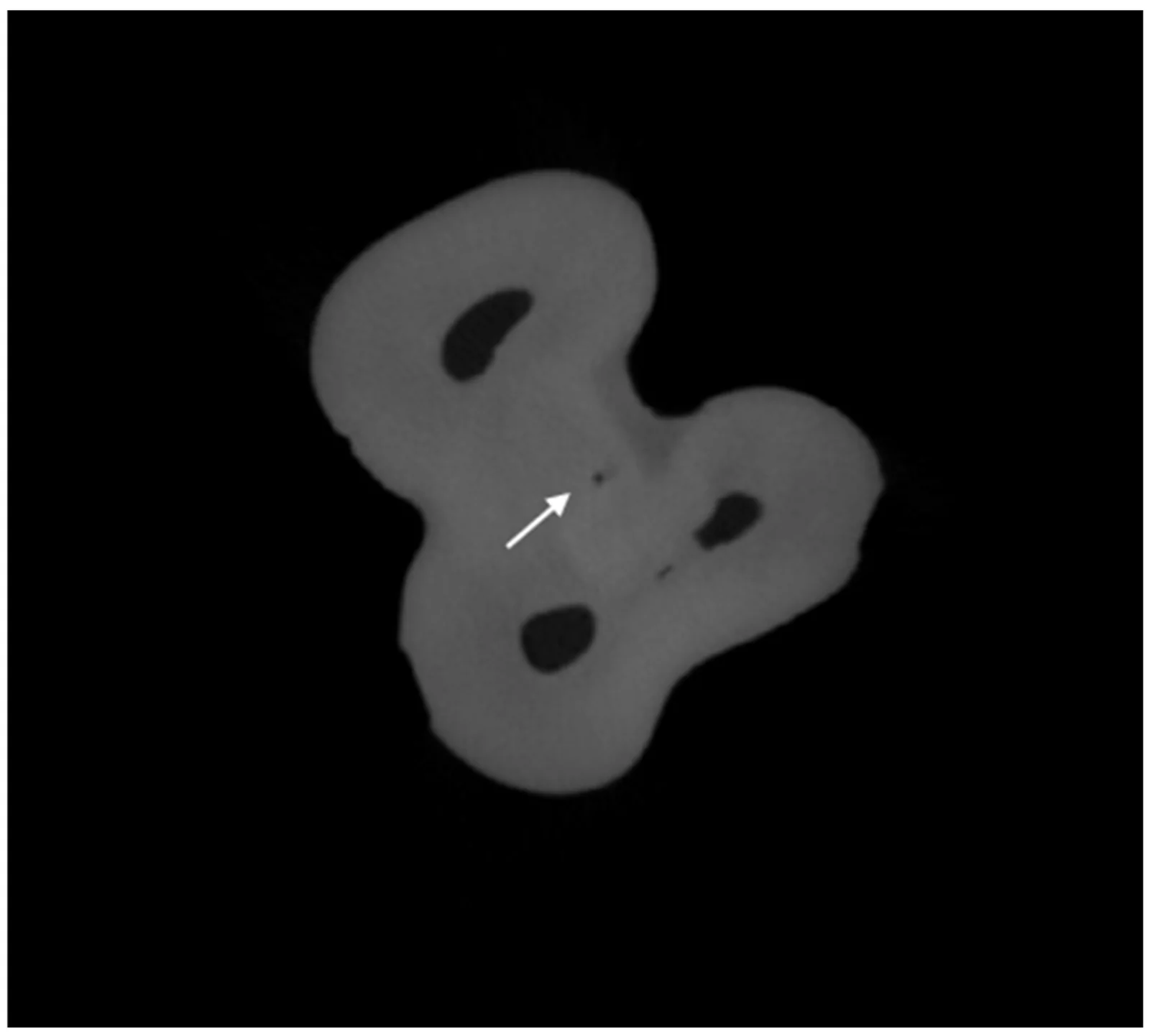
Accessory canal (arrow) in the furcation area in first primary upper molar.

The furcation structure thickness was significantly higher in second molars (median: 1.50 mm/1.46–1.55 mm) compared to first molars (median: 1.30 mm/1.24–1.33 mm) (*p* < 0.001). The rank-biserial correlation indicated large effect, reflecting minimal overlap between the distributions of the two molar groups. Maximum dentin thickness was significantly higher in second molars (median: 1.86/1.76–1.96 mm) than first primary molars (median: 1.53/1.51–1.62 mm, *p* = 0.003). Similarly, furcation dentin volume was significantly higher in second primary molars (80.8 mm^3^, 72.9–84.5) than in first primary molars (46.21 mm^3^, 38.4–46.7) (*p* < 0.001). These scanning parameters allowed for the detection of accessory canals in the furcation area in only 15.79% of molars (*n* = 6, Table 1, Figure 6).

## 4. Discussion

The present study aimed to quantitatively evaluate and compare the dentin mineral density, as well as furcation structure thickness, and maximum thickness and volume, of primary first and second molars using high-resolution micro-computed tomography. A key observation of this study is the significantly lower mineral density in the furcation region compared to the coronal dentin in both first and second primary molars (Figure 2); thus, the null hypothesis was rejected. This pattern suggests that the furcation area is structurally less mineralized, which may be related to its developmental characteristics and functional adaptation.

Furthermore, second primary molars showed significantly higher mineral density than first primary molars in both the crown and furcation regions (Figure 2). These compositional differences were accompanied by greater furcation structure thickness (1.50 mm in second molars vs. 1.30 mm in first molars) and substantially larger furcation dentin volume (80.8 mm^3^ vs. 46.2 mm^3^, respectively) in second molars (Figure 3 and Figure 4). Clinically, this lower mineralization may contribute to the higher susceptibility of the furcation area to pathological processes such as infection spread during pulpal inflammation.

The methodological approach used in this study followed a highly standardized micro-CT protocol. All teeth were scanned with the same settings on a SkyScan 1272 system (87 kV, 114 μA, 15 μm isotropic voxel size, 1 mm aluminium filter, full 360° rotation at 0.4° steps with four-frame averaging). Mineral density calibration was achieved using hydroxyapatite phantoms of 0.3 and 1.25 g/cm^3^ scanned under identical conditions, which allowed us to establish a linear relationship between grey values and mineral density. Consistent ROI definition for coronal and furcation dentin, combined with global thresholding guided by histogram analysis and selective manual corrections, helped minimize variability in segmentation. This level of standardization is particularly important in micro-CT research on dental tissues. As Tosco et al. have pointed out in their work on quantitative analysis of dental hard tissues and restorative interfaces, careful control of scanning parameters, phantom calibration, thresholding strategies, and precise ROI selection is crucial for obtaining reliable three-dimensional reconstructions and comparable quantitative results [17].

The mineral density of primary dentin assessed with micro-CT analysis has been shown to vary across tooth types [19]. In maxillary anterior teeth, it ranged from 0.68 to 2.00 g/cm^3^ (mean: 1.31 g/cm^3^), while in maxillary posterior teeth it ranged from 0.91 to 1.88 g/cm^3^ (mean: 1.43 g/cm^3^). In mandibular anterior teeth, the range was 0.14–2.08 g/cm^3^ (mean: 1.24 g/cm^3^), and in mandibular posterior teeth it was 0.87–2.20 g/cm^3^ (mean: 1.45 g/cm^3^) [19]. The findings in the present study are broadly with these data, as the mineral density values obtained in the present study ranged between 1.84 and 1.96 g/cm^3^ for primary molars.

Previous studies have also demonstrated that mineral density in primary dentin gradually decreases from the dentin-enamel junction toward the apex [19]. Furthermore, the observed lower mineral density in the furcation region in the present study is consistent with this gradient, as the furcation area is located closer to the pulp. This is supported by mechanical data, where hardness and elastic modulus decrease toward the pulpal region. Angker et al. showed that dentin nearest the pulp wall has significantly lower hardness and elastic modulus compared to dentin closer to the dentin-enamel junction [2]. This gradient is associated with reduced mineral content and increased carbonate substitution. Similarly, Torres et al. reported higher carbonate content in primary teeth compared to permanent teeth, which may influence both mechanical properties and acid resistance [16].

The findings of the present study are also partially consistent with those reported by Duarte et al., who used micro-CT to characterize the furcation area of primary molars [20]. They observed that all primary molars had resorption gaps and that 22.3% had accessory canals, highlighting the structural complexity of this region. These features may further contribute to the vulnerability of the furcation area. Interestingly, Duarte et al. reported that “mean grey values of the furcation area were higher than those of sound root dentine,” suggesting slightly higher microradiographic density [20]. In contrast, the present study, based on calibrated mineral density measurements (g/cm^3^), demonstrated lower mineral density in the furcation compared to the crown, which may reflect differences between true mineral quantification and grey value-based assessments.

The findings in this study regarding the lower mineral density and thinner structure in the furcation region of primary molars complement the morphological observations reported by Fumes et al., who used micro-CT to evaluate root canal anatomy in primary molars [18]. They described flat-shaped canals in the apical third of mandibular molars and ribbon- or oval-shaped canals in maxillary molars, highlighting the anatomical complexity of primary molar roots. The present study observed significant differences in furcation dentin thickness and volume between first and second primary molars, with first molars showing thinner and smaller furcation structures. These combined mineral density and morphometric findings further underscore the structural vulnerability of the furcation area, particularly in first primary molars. The lower micro-CT-derived mineral density and reduced furcation dentin dimensions observed in first primary molars suggest that this region may require increased attention during operative procedures involving the pulpal floor, including deep caries excavation, pulpotomy, pulpectomy, and restoration of extensive carious lesions. Preservation of dentin in the furcation region may be particularly important when treating first primary molars because of their comparatively smaller dentin volume and lower mineral-density estimates. Nevertheless, the present study only evaluated anatomical and micro-CT-derived mineral-density characteristics under in vitro conditions. It did not assess dentin mechanical properties, permeability, bacterial penetration, or clinical treatment outcomes.

In primary molars, the short distance from the pulpal floor to the furcation (approximately 1.7 mm), combined with lower mineralization in this area, may contribute to increased vulnerability during caries progression and a higher risk of furcation involvement or iatrogenic perforation [4]. Dabawala et al. confirmed that this distance remains consistently around 1.7 mm across primary molars, representing a critical anatomical limitation [4]. In addition, Duarte et al. reported a mean furcation dentin thickness of approximately 2.3 mm, which is slightly higher than the values observed in the present study, but still supports the concept of a relatively thin and clinically sensitive region [20]. It should be noted, however, that Duarte et al. assessed dentine mineralization using mean grey values at the furcation area compared with other regions of root dentine after micro-CT scanning and reconstruction with ImageJ software (NRecon v.1.6.6; Bruker, Kontich, Belgium). In contrast, the present study used hydroxyapatite calibration phantoms scanned under the same parameters to establish a linear calibration curve, allowing for conversion of grey values into hydroxyapatite-equivalent mineral density. Despite these methodological differences, both studies consistently demonstrate reduced mineralization (or lower grey values indicative of lower densities) of the furcation dentin compared with surrounding dentin, supporting the increased susceptibility of this region. The depth of demineralization in carious primary dentin can reach approximately 1100 μm, which is often greater than what is clinically apparent, further emphasizing the susceptibility of this area [21].

Morphologically, the significantly thicker furcation structure and larger dentin volume in second molars provide a greater physical buffer between the pulp chamber and the interradicular area. Diéguez-Pérez et al. reported considerable anatomical variability between molar types, with mandibular first molars showing the highest risk of perforation in the furcation area [1]. These findings align with the present results and may help explain why first primary molars are generally more prone to procedural complications. Such anatomical differences, together with variations in pulp chamber size and root canal morphology, support consideration of tooth-specific clinical approaches.

From a clinical perspective, the reduced mineral density in the furcation region has important implications for caries progression and treatment outcomes. Primary teeth are characterized by thinner dentin, larger pulp chambers, and higher tubule density compared to permanent teeth. Lenzi et al. demonstrated significantly higher tubule density in primary teeth (124,329 ± 43,594 mm^2^ vs. 45,972 ± 21,098 mm^2^), which facilitates faster progression of caries toward the pulp [22]. Lower mineralization may further reduce structural resistance and increase dentin permeability, potentially allowing infection to spread more easily into the interradicular region.

Regarding dentine mineral concentration, the results are consistent with those of Mijan et al., who evaluated mineral density in primary molars managed by different protocols using micro-CT with hydroxyapatite phantoms [13]. They reported hypermineralized areas underneath ART restorations and higher mineral concentrations in dentine subjacent to cleaned open cavities compared to untreated ones. The calibrated mineral density measurements in the present study similarly demonstrated lower mineralization in the furcation region and support the potential remineralization capacity of dentine when cavities are properly managed.

The presence of accessory canals has been investigated in numerous in vitro studies using different methods such as scanning electron microscopy, light microscopy and micro-CT and their prevalence ranged between 57% and 83% [23]. Additionally, anatomical studies have shown that 42.7% of primary molars present foramina in the furcation region [5]. Duarte et al. reported accessory canals in 22.3% of cases, with 19.5% showing direct communication between the pulp chamber and furcation [20]. The differences with the results from this study might be explained by the differences in methodology, imaging technique or voxel resolution, which may not have been sufficient to reliably detect very small canals, as well as the strict inclusion criteria regarding root resorption.

## 5. Limitations

The study has certain limitations that should be considered. The sample size, although statistically justified, remains relatively limited and may not fully represent the anatomical and biological variability in the pediatric population. Additionally, maxillary and mandibular molars were analyzed together despite known morphological differences between arches. Part of the observed variability in furcation morphology and dentin volume may be related to arch-specific anatomical characteristics. Thus, the present findings should therefore be interpreted as primarily morphometric comparisons between first and second primary molars within the studied sample. Future studies with larger and balanced samples should perform stratified analyses according to both molar type and dental arch. Additionally, some assessments such as mineral density analysis and maximum dentin thickness in the furcation area depended on manual segmentation, ROI selection and measurements, which may introduce operator-dependent variability. Although intra-observer reproducibility was excellent, inter-observer reliability was not assessed. The voxel resolution used in this study may have limited the detection of extremely fine accessory canals, potentially leading to an underestimation of their true prevalence. Finally, the study was conducted on extracted teeth under in vitro conditions, which may not completely replicate the complex biological environment present in vivo. Factors such as the degree of physiological resorption and individual differences in mineralization were controlled for as much as possible but cannot be entirely eliminated. Although effect-size estimates and confidence intervals provide information regarding the magnitude and precision of the observed anatomical differences, established thresholds for clinically meaningful differences in primary molar dentin mineral density and furcation morphometric parameters are currently unavailable. Therefore, the clinical relevance of the observed effect magnitudes should be interpreted cautiously and requires validation in studies directly evaluating mechanical properties, disease progression, and treatment outcomes.

## 6. Conclusions

This micro-CT analysis demonstrated that dentin mineral density is significantly lower in the furcation region compared to the coronal dentin in primary molars. Second primary molars exhibited higher mineral density, greater furcation structure thickness, and larger furcation dentin volume than first primary molars. These regional and tooth-type differences highlight the furcation area as a structurally and compositionally vulnerable zone, particularly in first primary molars. The findings suggest that such anatomical and mineralization variations may contribute to increased clinical risk during caries progression or endodontic procedures and support the consideration of tooth-specific approaches in the management of primary molars. Further clinical studies are needed to evaluate the direct impact of these morphological features on treatment outcomes and long-term prognosis.

## Data Availability

The data presented in this study are available in this article. Raw and processed micro-CT datasets are available from the corresponding author upon reasonable request.

## Abbreviations

The following abbreviations are used in this manuscript:

Micro-CT	Micro-computed tomography
MD	Mineral density
ROI	Region of interest
